# The impact of health education on the prevalence of faecal-orally transmitted parasitic infections among school children in a rural community in Cameroon

**DOI:** 10.4314/pamj.v8i1.71153

**Published:** 2011-02-04

**Authors:** Henri Lucien Fouamno Kamga, Dickson Shey Nsagha, Mary Bi Suh Atanga, Anna Longdoh Njunda, Jules Clement Nguedia Assob, Peter Nde Fon, Solange Akwi Fomumbod

**Affiliations:** 1 Department of Medical Laboratory Sciences, Faculty of Health Sciences, University of Buea, Cameroon; 2 Department of Public Health and Hygiene, Faculty of Health Sciences, University of Buea, Cameroon; 3 Department of Nursing, Faculty of Health Sciences, University of Buea, Cameroon; 4 Medicine programme, Faculty of Health Sciences, University of Buea, Cameroon

**Keywords:** Intestinal parasites, parasitic infections, health education, Cameroon

## Abstract

**Background>:**

Faecal-orally transmitted parasites are those parasites which are spread through faecal contamination of food and drinks. Infections with these parasites are among the most common in the world being responsible for considerable morbidity and mortality, especially in children. This study was carried out to determine the impact of health education on the prevalence of faecal-orally transmitted parasitic infections among primary school children in a typical African rural community.

**Methods:**

An intervention study was conducted in two villages in the South-West Region of Cameroon. A total of 370 volunteer pupils aged between 5-15 years were enrolled in the study out of which 208 were from Kake II (experimental arm) and 162 from Barombi-Kang (control arm). The research was conducted in two phases. In phase 1, stool samples were collected from all participants and analyzed using the formol-ether concentration technique and health education was given to the pupils in the experimental village but not in the control village. Phase 2 was conducted six months later during which only stool samples were collected and analyzed from both villages.

**Results:**

Before health education intervention (phase1) faecal-orally transmitted parasites were present in 106 (50.9%) stool specimens collected in Kake II and in 84 (51.5%) of those collected in Barombi-kang. The difference in prevalence between these two villages was not significant (P>0.05). After health education intervention (phase2), 56 (26.9%) stool specimens were positive for faecal-oral parasite in Kake II and 92 (54.7%) in Barombi-kang, and the difference in prevalence between these two villages was statistically significant (P0.05). The change in the prevalence of infection was significant in Kake II (50.9% vs. 26.9%, P0.05). Hence, health education applied in the experimental village was responsible for the drop in the prevalence observed, especially among pupils infected with Ascaris lumbricoides (24.9% vs. 3.4%, P=0.004)

**Conclusion:**

Health education through the framework of schools can be used as a strategy for the control of faecal-orally transmitted parasitic infections among children in African rural communities.

## Background

Intestinal parasitic infections (IPIs) are among the most prevalent infections in humans in developing countries and are responsible for considerable morbidity and mortality. Most of them are transmitted by the faecal-oral route. In general, situations involving unhygienic conditions promote transmission [1]. These infections are globally endemic and have been described as constituting the greatest single worldwide cause of illness and disease [2]. They are associated with poor hygiene and lack of access to safe water [3]. Food handlers play an important role in their transmission [4]. Ignorance is also a contributing factor to transmission especially among people living in rural areas where level of awareness is relatively low [5]. Like the majority of the parasitic diseases, these infections are influenced by human behaviour especially their hygienic practices, and failure to take advantage of available screening services or comply with treatment [6].

In Cameroon, IPIs are recognized by the Ministry of Public Health to be an important public health problem ranking second to malaria [7]. There has been periodic de-worming in most endemic areas in the country, but studies suggest that there is a high rate of re-infection [8]. A change in hygienic behaviour is advocated to reduce these infections. Health education, especially in rural communities where the level of awareness is quite low and sanitary conditions poor [9] could contribute to the reduction of the prevalence of infection: This issue is addressed in this study.

## Methods

Ten villages in the South-West Region of Cameroon were grouped in pairs. The grouping was based on the fact that each pair was made of 2 rural communities sharing the same social, geographical and climatic features. The pair comprising Kake II and Barombi-Kang was randomly selected among five. An intervention study was conducted from January 2010 to July 2010 in two primary schools in the 2 selected villages (Kake II and Barombi-kang). They are separated by a distance of about 30 Km. The former village served as the experimental school, whilst the latter served as the control (random selection). The two villages are typical African rural communities. There is no pipe-borne water, electricity, or drainage system. Each of the villages has a government primary school, but no secondary school. The inhabitants are farmers practicing peasant farming and petty trading.

Prior to the start of this study, permission was sought from the school authorities and parents were informed about the purpose, objectives and benefits of the study, as their involvement was a key factor for its success. They were made to understand that it was not a school obligation to take part in the study, neither was it a prerequisite for accessing publicly available health facilities. Written informed parental consent forms were distributed one week prior to the beginning of sample collection. An ethical clearance was obtained from the Regional Delegation of Health of the South West Region in Buea (Ref.R11/MPH/SWP/RDPH/FP-R/5341/94).

**Phase 1**

After registration, stool samples were collected in well labelled 50 ml screw-cap vials and then transported to the Kumba district hospital laboratory. Samples were collected from volunteer pupils aged between 5-15 years. Stool specimens were analyzed using the formol-ether concentration technique as already described elsewhere [10]. All slides were read within 24 h of preparation to avoid the degeneration of Ancylostoma sp. eggs. Following sample collection, health education was given to the pupils in Kake II school (experiment), and it was aimed at promoting and reinforcing health behaviour with particular reference to the need to encourage aspects of personal hygiene relevant to the control of faecal-orally transmitted parasitic infections. Focus group discussions were held in the school using pictorial cards. Basic messages communicated were: what faecal-orally transmitted parasites are, how the infections are acquired, what the signs and symptoms are, what can be done to prevent these parasites, and the importance of visiting the health centre for treatment. In Barombi-Kang (control area), only the baseline data were collected without any health education.

**Phase 2**

This phase was conducted in July 2010, 6 months after the end of the first intervention and consisted of collection and analysis of samples and detection of cysts, ova and larvae of parasites. Pupils from the control area (Barombi-Kang), however, received health education at the end of the study.

Data was entered using Epi-Info 6.04 (CDC) and analyzed using the Statistical Package for Social Sciences version 17.0 (SPSS Inc. 2008). The Chi-Square test was used to compare proportions before and after the health education intervention at significant level of 0.05.

## Results

A total of 370 pupils volunteer aged 5 to 15 years participated in this study. They were from two government primary schools in two villages of the Kumba district in the South West Region of Cameroon. The two villages were Kake II (Experimental arm) and Barombi-Kang (control arm). Stool samples were collected in 2 phases. In the first phase, 370 samples were collected of which 208 (56.2%) were from Kake and 162 (43.8%) from Barombi-Kang. In the second phase, 368 samples were collected of which 208 (56.5%) were from Kake and 160 (43.5%) from Barombi-Kang.

The Prevalence of faecal-orally transmitted parasites in Kake II and Barombi-Kang villages before and after the health education intervention is shown in Table 1. Prior to health education intervention (phase1) parasites were present in 106 (52.7%) stool specimens collected in Kake II, the experimental village and in 84 (51.5%) of those collected in Barombi-kang, the control village. The difference in prevalence between these two villages was not significant (P>0.05). After the health education intervention (phase2), 56 (26.9%) stool specimens were positive for parasites in Kake II and 92 (54.7%) in Barombi-kang, and the difference in prevalence between these two villages was statistically significant (P< 0.001). The change in the prevalence of infection was significant in Kake II (50.9% vs. 26.9%, P0.05).

The Prevalence of faecal-orally transmitted parasites in Kake II and Barombi-Kang villages according to age before and after health education intervention is shown in Table 2. There was no significant difference between the prevalence of faecal-orally transmitted parasites and the ages of infected pupils (P>0.05).

Table 3 shows the prevalence of different species of faecal-orally transmitted parasites in Kake II (experimental arm) and Barombi-Kang (control arm), before and after the health education intervention. In Kake II, the change in the prevalence of parasites was more significant for *Ascaris lumbricoides* (24.9% vs. 3.4%, P*Entamoeba coli* (12.9% vs. 6.5%, P*Trichuris trichiura* (22.4% vs. 12.5%, P=0.004) and *Entamoeba histolytica* (6.0% vs. 1.9%, P=0.035). In Baombi-Kang, there was no significant change in the prevalence of any of the faecal-orally transmitted parasites detected.

## Discussion

This study was targeted at school children as they are mostly the heavily infected individuals with faecal-orally transmitted parasites and the most at risk of developing severe disease [11]. School children are also likely to contribute most to transmission [9] and they are readily accessible through the framework of schools. The prevalence of faecal-orally transmitted parasites in Kake II (experimental arm) and Barombi-Kang (control arm) villages before health education intervention were 52.7% and 51.5% respectively. This result is close to that reported by Hamit et al. [12] in Chad (51%) and Mazigo et al. [13] in Tanzania (57.1%), but higher than that reported by Kanoa et al. [14] in Gaza and Uneke et al. [15] 146 in Nigeria. The differences could be attributed to geographical differences, but more likely to differences in behavioural and hygienic factors among the school children.

Comparing the prevalence of faecal-orally transmitted parasites obtained before and after health education intervention, we observed that in Kake II where health education was carried out, there was a significant drop in the prevalence of the parasites. In Barombi-Kang, in which no health education was given, the change was not significant. The significant change in prevalence in the experimental village cannot be attributed to social or seasonal variation; the two villages share the same social, geographical and climatic features. Only health education applied in the experimental villages could have accounted for the observed decrease.

Comparing the prevalence of the faecal-orally transmitted parasites according to age in the two villages studied, one can deduce that health education had a closely similar impact in all the age groups between 5 and 15 years. It is an indication that the observed decrease in prevalence in the experimental village evenly affected children of all ages. This observation differs from that of Kamga [16] who found a greater drop of prevalence among children of the 10-14 age group following health education in a similar study on urinary schistosomiasis carried out in the North of Cameroon. In that study, the sample population included primary and secondary school children from different institutions while the present study involved only primary school pupils of the same school. Due to the likelihood of interaction among the pupils, it is believed that some children might have served as health educator messengers, as they were able to exert their influence on other children of any age (in and out of school) and on their family members.

Comparing the prevalence of different parasites in the experimental village where health education was implemented, there was a significant drop in *Ascaris lumbricoides, Entamoeba coli, Trichuris trichuira and Entamoeba histolytica*. It is likely that health education have had a significant impact on recreational high-risk behaviour of the pupils and also on their willingness to take advantage of available screening services. This proves that children who are conversant with the health knowledge of the disease are more knowledgeable of the consequences of many attitudes and practices than those who have not been exposed to such an opportunity. It follows that through health education, a child is exposed to health ideals which enhance his/her health. However, the low infestation rate recorded during the second phase of our study in the experimental village could also be due to self-medication on the part of the children. In Cameroon albendazole, Mebendazole and metronidazole which are known to give good efficacy on those parasites [11,17,18] can easily be obtained from street vendors at low cost. This tendency for people to purchase drugs from street vendors and the consequent repeated and indiscriminate use of medication without medical supervision, in addition to mask the results of parasitological assays and consequently the prevalence; may induce the long term resistance of parasites to these drugs. This has great consequences on the transmission of these diseases [19] and can have a direct relationship with IPIs morbidity.

## Conclusion

This study has shown that in rural areas such as kake II, lack of adequate knowledge of faecal-orally transmitted parasites and long standing traditional practice and beliefs are barriers to improving the faecal-orally transmitted parasites situation. Also, the improved level of knowledge on faecal-orally transmitted parasites and related practices had a positive impact in reducing the prevalence of the infection. Therefore, health education through the framework of schools can be used as a national control strategy of faecal-orally transmitted parasitic infections among school children.

## Competing interests

The authors declare no competing interests.

## Acknowledgments

We thank the People of Kake II and Barombi-Kang for their cooperation and assistance in the data collection, and the staff members of the Kumba District Hospital for their collaboration in the laboratory investigation.

## Authors contributions

HLF Kamga designed the study, analysed the data, drafted the manuscript and substantially revised it. DS Nsagha contributed in the design, write-up and substantially revised the manuscript. MBS Atanga, AL Njunda, JCN Assob and P Nde Fon participated in the write-up and substantially revised the manuscript for academic content, and SA Fomumbod participated in the laboratory investigation. All authors read and approved the final version of the manuscript.

## Figures and Tables

**Table 1:** Prevalence of faecal-orally transmitted parasites in Kake II (experimental arm) and Barombi-Kang (control arm) before and after health education intervention

Study area	Number (%)infected with parasites	
	Before health education intervention	After health education intervention
	**No (%)**	**No (%)**
Kake II (experimental arm)*	106 (50.9)	56 (26.9)
Barombi-Kang (control arm)	84 (51.5)	92 (57.5)
**Total**	**190(51.3)**	**148 (40.2)**

* P< 0.005

**Table 2:** Prevalence of faecal-orally transmitted parasites according to age in Kake II (experimental arm) and Barombi-Kang (control arm) before and after health education intervention

Study area	Age	Number (%) of samples infected with parasites	
		before health education intervention No (%)	after health education intervention No (%)
Kake II (experimental arm)	5-9	30(14.4)	19(9.1)
	10-12	65(31.2)	28(13.4)
	12-15 Total	11(5.3)	9(4.3)
	Total	106(50.9)	56(26.9)
Barombi-kang (Control arm)	5-9	32(18.8)	29(17.2)
	10-12	40(23.5)	41(24.4)
	12-15	12(7.1)	22(13.1)
	**Total**	**84(49.4)**	**92(54.7)**

**Table 3:** Prevalence of different species of faecal-orally transmitted parasites in Kake II (experimental arm) and Barombi-Kang before (control arm) before and after health education intervention

Parasite species	Number (%) of infected stool samples in Kake II (experimental arm)		Number (%) of infected stool samples in Barombi-Kang(control arm)	
	Before health education	After health education	Phase 1 intervention	Phase 2 intervention
A.lumbricoides*	50 (24.9)	7 (3.4)	28 (17.3)	29 (18.1)
T.trichiura*	47 (22.4)	26 (12.5)	18 (11.1)	16 (0.1)
*E.vermicularis*	3 (1.5)	4 (1.9)	10 (6.2)	9 (5.6)
*Ancylostomasp.*	8 (4.0)	7 (3.4)	12 (7.4)	18 (11.2)
*H.nana*	0 (0.0)	1 (1.6)	1(0.6)	2 (1.2)
E.histolytica*	12 (6.0)	4 (1.9)	16 (9.8)	15 (9.3)
Entamoebacoli*	29 (12.9)	8 (6.5)	21 (12.9)	20 (12.5)
*G.lamblia*	3 (1.5)	2 (1.0)	6 (3.7)	7 (4.3)
*B.coli*	1 (0.5)	1 (0.5)	1 (0.6)	1 (0.6)

* P< 0.005 in Kake II (experimental arm)
